# Phycocyanobilin Attenuates Oligomerized Amyloid β-Induced Neuronal Senescence Through SIRT1-Associated Mechanisms

**DOI:** 10.3390/nu18152579

**Published:** 2026-08-06

**Authors:** Mei Chou Lai, Yu-Shun Tzeng, I-Min Liu

**Affiliations:** 1 Department of Pharmacy and Master Program, College of Pharmacy and Health Care, Tajen University, Pingtung County 90741, Taiwan; meei@tajen.edu.tw; 2 Department of Medicine, College of Medicine, National Cheng Kung University, Tainan 70101, Taiwan; i54106318@gs.ncku.edu.tw

**Keywords:** amyloid-β, phycocyanobilin, algae nutritive compound, senescence, SH-SY5Y cells

## Abstract

**Background/Objectives:** Alzheimer’s disease (AD) is associated with amyloid-β (Aβ)-induced neuronal injury, oxidative stress, inflammatory activation, and cellular senescence. Phycocyanobilin (PCB), an algae nutritive compound, has shown neuroprotective potential, but whether it attenuates Aβ-induced neuronal senescence remains unclear. This study investigated the protective effects of PCB against Aβ_1–42_ oligomer-induced senescence-like alterations in SH-SY5Y cells and examined the involvement of sirtuin 1 (SIRT1) signaling. **Methods:** Differentiated SH-SY5Y cells were pretreated with PCB (50 μmol/L) in the presence or absence of EX527 (10 μmol/L) for 24 h, followed by exposure to Aβ_1–42_ oligomers (20 μmol/L) for an additional 24 h. Cell viability, lactate dehydrogenase (LDH) release, senescence-associated β-galactosidase (SA-β-gal) staining, senescence-associated heterochromatin foci (SAHF)-associated di-/tri-methylated histone H3 lysine 9 (H3K9me2/3) fluorescence, senescence-associated secretory phenotype (SASP)-related cytokines, phosphorylated histone H2AX (γ-H2AX) accumulation, Ki67 expression, p53/p21/p16 signaling, and silent information regulator 1 (SIRT1) expression and activity were assessed using cell counting kit-8 assay, LDH assay, enzyme-linked immunosorbent assay, immunofluorescence staining, quantitative real-time PCR analysis, Western blotting, and fluorometric enzymatic activity assay. **Results:** Aβ_1–42_ oligomers reduced cell viability, increased LDH release, promoted SA-β-gal positivity, enhanced H3K9me2/3 and γ-H2AX signals, elevated IL-1β, IL-6, and IL-8 levels, suppressed Ki67 expression, and upregulated p53, p21, and p16. PCB pretreatment markedly attenuated these cytotoxic, senescence-associated inflammatory, and DNA damage-related responses while restoring SIRT1 mRNA, protein expression, and enzymatic activity. EX527 partially reversed these protective effects. **Conclusions:** PCB attenuates Aβ_1–42_ oligomer-induced neuronal senescence-like alterations, at least partly through SIRT1-associated regulation, supporting its potential as a naturally derived anti-senescent neuroprotective compound for AD-related neuronal aging.

## 1. Introduction

Alzheimer’s disease (AD) is a progressive age-associated neurodegenerative disorder and the leading cause of dementia in older adults [1]. Clinically, AD manifests as gradual memory impairment, cognitive decline, behavioral disturbances, and personality changes, reflecting the progressive disruption of neuronal circuits involved in learning, memory, and higher cognitive functions [2]. At the neuropathological level, AD is characterized by synaptic dysfunction and neuronal loss, together with extracellular amyloid plaques and intracellular neurofibrillary tangles [3]. Among these neuropathological features, amyloid-β (Aβ) has long been recognized as a major contributor to AD pathogenesis. In particular, the aggregation-prone Aβ_1–42_ species is closely associated with synaptic damage, oxidative and mitochondrial stress, inflammatory activation, and neuronal vulnerability [4]. Rather than representing inert deposits, soluble Aβ oligomers and intracellular Aβ pools are increasingly viewed as bioactive pathogenic species that can initiate disease-relevant downstream processes, including endolysosomal dysfunction, proteostatic imbalance, inflammatory signaling, and progressive neuronal impairment [5]. Although amyloid-centered therapeutic strategies have advanced the disease-modifying treatment landscape of AD, their clinical benefits remain limited, suggesting that the mechanisms linking Aβ-related proteotoxic stress to persistent and self-amplifying neuronal injury require further clarification [6].

In this context, cellular senescence has emerged as an aging-related stress program that may link Aβ-associated molecular injury to sustained neurodegenerative signaling [7,8]. Senescent cells exhibit senescence-associated β-galactosidase (SA-β-gal) activity, persistent DNA damage responses, chromatin remodeling, stable cell-cycle arrest, and the senescence-associated secretory phenotype (SASP) [9]. Through the secretion of proinflammatory cytokines, chemokines, proteases, and other stress mediators, SASP-positive cells may reshape the surrounding microenvironment and propagate senescence-like stress to neighboring cells, thereby amplifying neuroinflammatory and degenerative responses [10]. At the molecular level, Aβ-induced oxidative stress, mitochondrial dysfunction, and proteostatic imbalance may converge on DNA damage response pathways and activate p53/p21- and p16-associated senescence programs, further promoting inflammatory amplification and reduced neuronal resilience [11]. Silent information regulator 1, also known as sirtuin 1 (SIRT1), is a nicotinamide adenine dinucleotide (NAD)-dependent deacetylase that regulates stress resistance, DNA repair, mitochondrial homeostasis, inflammatory control, and senescence-related signaling [12]. Reduced SIRT1 activity has been associated with enhanced cellular stress and senescence-associated responses, whereas preservation of SIRT1 function may help restrain p53-related growth arrest and maintain neuronal homeostasis [13]. Therefore, therapeutic strategies that extend beyond amyloid clearance and target SIRT1-associated oxidative stress, inflammatory signaling, and senescence-like responses may provide a complementary approach for mitigating AD-related neuronal dysfunction [10,14].

Algae-derived pigments and phycobiliprotein-related compounds have been reported to exert neuroprotective effects through multiple mechanisms, including reduction of reactive oxygen species, activation of antioxidant pathways, suppression of neuroinflammatory signaling, and preservation of mitochondrial function [15]. Phycocyanobilin (PCB), a linear tetrapyrrolic chromophore derived from C-phycocyanin in cyanobacteria and red algae, has attracted attention as a small bioactive molecule with free-radical scavenging activity and protective effects against oxidative neuronal injury [16]. Consistent with this profile, PCB has been shown to protect neuronal cells from glycation-associated injury by preserving cell viability and neurite integrity, suppressing RAGE/NOX4-related oxidative stress, attenuating endoplasmic reticulum stress, and inhibiting mitochondrial apoptotic signaling [17]. These pleiotropic actions suggest that PCB may serve as a nutritive compound with potential relevance to neurodegenerative conditions characterized by oxidative stress, inflammation, and impaired cellular homeostasis [15]. Nevertheless, whether PCB can attenuate Aβ-induced cellular senescence remains insufficiently defined. Addressing this question is important because evidence of an anti-senescent effect would broaden the biological significance of PCB beyond its established antioxidative and cytoprotective properties.

The present study aimed to investigate whether PCB protects neuronal cells against Aβ_1–42_-induced injury and senescence-associated alterations. To this end, we established an in vitro AD-related neuronal injury model using SH-SY5Y cells exposed to Aβ_1–42_ and evaluated the protective effects of PCB on cytotoxicity, senescence-associated phenotypes, SASP-related inflammatory responses, DNA damage, proliferative capacity, and p53-p21/p16 signaling. Furthermore, to determine whether SIRT1 contributes to the protective effects of PCB, we assessed SIRT1 expression and enzymatic activity and employed the selective SIRT1 inhibitor EX527 (Selisistat) as a pharmacological tool [18]. Through this approach, we aimed to determine whether PCB attenuates Aβ-induced neuronal senescence via SIRT1-associated regulatory mechanisms, thereby providing mechanistic insight into its relevance as an algae-derived nutritive compound for AD-related neuronal aging.

## 2. Materials and Methods

### 2.1. Cell Culture and Neuronal Differentiation

Human SH-SY5Y neuroblastoma cells (CRL-2266; ATCC, Manassas, VA, USA) were employed as a neuronal-like in vitro model [19]. Cells were propagated in DMEM/F12 medium (Cat# SLM-243-B; Sigma-Aldrich, St. Louis, MO, USA) containing 10% fetal bovine serum (FBS; Cat# F2379; Sigma-Aldrich, St. Louis, MO, USA), 1% non-essential amino acids (Cat# 11140050; Thermo Fisher Scientific, Waltham, MA, USA), and penicillin–streptomycin at final concentrations of 100 U/mL and 100 µg/mL, respectively (Cat# 15140148; Thermo Fisher Scientific, Waltham, MA, USA). Cultures were maintained at 37 °C in a humidified atmosphere with 5% CO_2_. For neuronal induction, SH-SY5Y cells were plated in 100-mm culture dishes at 1 × 10^6^ cells per dish and allowed to adhere in complete growth medium. Once the cells reached approximately 40–50% confluence, differentiation was initiated by adding all-trans retinoic acid (RA; 10 µmol/L; Cat# R2625; Sigma-Aldrich, St. Louis, MO, USA). During the differentiation period, the RA-containing medium was replaced at regular intervals. Differentiation status was evaluated morphologically by observing neurite development, which was defined as the extension of primary processes from the cell body. When neurite quantification was required, neurite outgrowth was assessed manually and analyzed using ImageJ software (version 1.6.0; NIH, Bethesda, MD, USA). After five days of RA exposure, differentiated cells were carefully detached with 0.05% trypsin prepared in phosphate-buffered saline (PBS, pH 7.4). The cells were then transferred to six-well plates at a density of 1 × 10^5^ cells per well for subsequent experiments. Experimental treatments were started only after the reseeded differentiated cells had attached and reached the appropriate confluence. All treatment procedures were performed after completion of the five-day RA differentiation protocol, based on previously described methods [17].

### 2.2. Preparation of Aβ_1–42_ Using an Oligomerization Protocol

Aβ_1–42_ was prepared using a previously described oligomerization protocol with slight modifications [20]. Amyloid-β_1–42_ peptide (Cat# ab120301; Abcam, Cambridge, UK) was first dissolved in 1,1,1,3,3,3-hexafluoro-2-propanol (HFIP; Cat# 105228; Sigma-Aldrich, St. Louis, MO, USA) with sonication to disrupt pre-existing aggregates and obtain a monomerized peptide preparation. The HFIP-treated peptide solution was aliquoted at 100 μg per tube, and HFIP was allowed to evaporate overnight to form a thin peptide film. The dried peptide aliquots were then stored at −20 °C until use. Prior to experiments, the peptide aliquots were re-treated with HFIP, vacuum-dried for 1 h to remove residual solvent, and dissolved in 10 mmol/L NaOH. After sonication, the solution was dried again, and the resulting peptide pellets were resuspended in 2 μL dimethyl sulfoxide (DMSO; Cat# 2650; Sigma-Aldrich, St. Louis, MO, USA). The peptide solution was then diluted with PBS to a final concentration of 1 μg/μL. To promote oligomer formation according to the reported protocol, the diluted peptide solution was incubated at 37 °C for 24 h and then used freshly for subsequent experiments to minimize variability in aggregation status. Therefore, the Aβ_1–42_ preparation used in this study is referred to as an oligomerization protocol-derived Aβ_1–42_ preparation.

### 2.3. Aβ_1–42_ Oligomer Exposure and Pharmacological Treatments

PCB (Cat# P2172; Sigma-Aldrich, St. Louis, MO, USA) was dissolved in PBS to prepare a 1 mmol/L stock solution, sterilized through a 0.2-μm syringe filter (Minisart^®^, Sartorius, Germany), and stored at 4 °C in the dark. EX527, a selective SIRT1 inhibitor (Cat# E7034; Sigma-Aldrich, St. Louis, MO, USA), was dissolved in DMSO to generate a 10 mmol/L stock solution. Before use, both stock solutions were freshly diluted with culture medium to the required working concentrations. The final DMSO concentration was kept below 0.1% (*v*/*v*), which has been reported to be non-cytotoxic [21]. Vehicle controls contained equivalent amounts of PBS and/or DMSO without active compounds.

To establish the Aβ_1–42_ oligomer-induced injury model, cells were exposed to Aβ_1–42_ oligomers at 5, 10, or 20 μmol/L for 12–48 h, based on previously reported Aβ neurotoxicity models [22]. Following viability screening, 20 μmol/L Aβ_1–42_ oligomers for 24 h was selected for subsequent experiments because it produced moderate and reproducible cytotoxicity while maintaining sufficient viable cells for evaluating PCB-mediated protection.

For protective experiments, cells were pretreated with PCB at 10, 30, or 50 μmol/L for 24 h before exposure to 20 μmol/L Aβ_1–42_ oligomers for another 24 h. These concentrations were selected according to previous evidence of PCB-mediated protection against glycation-associated neuronal injury [17]. To assess the involvement of SIRT1 signaling, cells were pretreated with 50 μmol/L PCB with or without EX527 at 10 μmol/L for 24 h before Aβ_1–42_ oligomer exposure [23]. PCB-alone and EX527-alone groups were also included to evaluate their potential cytotoxicity.

### 2.4. Measurement of Cell Viability

Cell viability was assessed using the Cell Counting Kit-8 assay (CCK-8; Cat# 96992; Sigma-Aldrich, St. Louis, MO, USA) [24]. SH-SY5Y cells were seeded in 96-well plates at a density of 5 × 10^3^ cells per well and allowed to attach overnight. Cells were then treated with Aβ_1–42_ oligomers at 5, 10, or 20 μmol/L for 12, 24, 36, or 48 h to evaluate concentration- and time-dependent Aβ_1–42_ oligomer-induced cytotoxicity. For protective experiments, cells were pretreated with PCB at 10, 30, or 50 μmol/L for 24 h before exposure to 20 μmol/L Aβ_1–42_ oligomers for an additional 24 h. To examine the involvement of SIRT1 signaling, cells were pretreated with PCB at 50 μmol/L in the presence or absence of EX527 at 10 μmol/L for 24 h, followed by exposure to 20 μmol/L Aβ_1–42_ oligomers for an additional 24 h. After treatment, 10 μL of CCK-8 reagent was added to each well, and the plates were incubated for 2 h at 37 °C. The absorbance of the resulting water-soluble formazan product was measured at 450 nm using a SpectraMax M5 microplate reader (Molecular Devices, Sunnyvale, CA, USA). Cell viability was normalized to the vehicle-treated control group, which was defined as 100%, and all experimental groups were expressed relative to this control.

### 2.5. Lactate Dehydrogenase (LDH) Release Analysis

LDH release was measured using an LDH assay kit (Cat# ab102526; Abcam, Cambridge, UK) as previously described [25]. Briefly, SH-SY5Y cells were seeded into 96-well plates and subjected to the indicated treatments. After treatment, culture supernatants were collected and centrifuged at approximately 120× *g* for 5 min at 4 °C to remove insoluble debris. The clarified supernatants were transferred to a new 96-well plate, and 50 μL of LDH reaction mixture was added to each well. After incubation at 37 °C for 1 h, absorbance was measured at 450 nm using a SpectraMax M5 microplate reader (Molecular Devices, Sunnyvale, CA, USA). Background absorbance was subtracted before analysis. LDH release was normalized to the vehicle-treated control group and expressed as fold change relative to control.

### 2.6. Measurement of SASP-Associated Cytokines by Enzyme-Linked Immunosorbent Assay (ELISA)

Following the indicated treatments, the concentrations of SASP-associated inflammatory cytokines, including interleukin (IL)-1β, IL-6, and IL-8, in culture supernatants were quantified using enzyme-linked immunosorbent assay (ELISA) kits following the protocols provided by the manufacturers. The IL-1β kit (Cat# ab100705) and IL-6 kit (Cat# ab222503) were obtained from Abcam (Cambridge, UK), while IL-8 was analyzed using a kit supplied by MyBioSource, Inc. (Cat# MBS286946; San Diego, CA, USA). Optical density was measured at 450 nm with a SpectraMax M5 microplate reader (Molecular Devices, Sunnyvale, CA, USA). Cytokine concentrations were then derived from the corresponding standard curves. To minimize the influence of treatment-related differences in cell number or cytotoxicity, cytokine values were adjusted according to the total protein content of each corresponding well. Total cellular protein was determined using a BCA protein assay kit (Cat# ab102536; Abcam, Cambridge, UK). The final cytokine results were reported as pg/mg protein.

### 2.7. Senescence-Associated β-Galactosidase (SA-β-Gal) Staining

Cellular senescence was assessed using a senescence β-galactosidase staining kit (Cat# 602010; Cayman Chemical, Ann Arbor, MI, USA) following a previously published methodology [26]. Briefly, cells cultured in six-well plates were gently washed with 2 mL of PBS and fixed with 1 mL of fixing solution per well for 15 min at room temperature. After removal of the fixative, cells were rinsed again with 2 mL of PBS, and 1 mL of SA-β-gal staining solution was added to each well. The plates were sealed to minimize evaporation and incubated at 37 °C in a CO_2_-free incubator for 16 h. SA-β-gal-positive senescent cells were identified by blue staining under a light microscope (Olympus CKX53; Olympus Corporation, Tokyo, Japan). For each condition, five randomly selected microscopic fields were evaluated, and the percentage of SA-β-gal-positive cells was calculated as the number of blue-stained cells divided by the total number of cells in each field.

### 2.8. Immunofluorescence Staining

Immunofluorescence staining was performed to evaluate senescence-associated heterochromatin remodeling, DNA damage, and cell-cycle-associated marker expression by detecting H3K9me2/3, γ-H2AX, and Ki67, respectively [27,28]. After treatment, cells were fixed with 4% paraformaldehyde for 15 min at room temperature, permeabilized and blocked for 1 h in PBS containing 5% normal goat serum (Cat# G9023; Sigma-Aldrich, St. Louis, MO, USA) and 0.3% Triton X-100. Cells were then incubated overnight at 4 °C with primary antibodies against di-/tri-methyl-histone H3 (Lys9) (6F12; Cat# 5327), γ-H2AX (Cat# 7632), or Ki67 (Cat# 62548), all from Cell Signaling Technology (Danvers, MA, USA). After washing, cells were incubated with Alexa Fluor^®^ 488-conjugated secondary antibody (Cat# 4408; Cell Signaling Technology, Danvers, MA, USA) for 1–2 h at room temperature in the dark, followed by DAPI counterstaining (Cat# 4083; Cell Signaling Technology, Danvers, MA, USA).

Fluorescence images were captured with an Olympus FV3000 confocal laser scanning microscope (Olympus Corporation, Tokyo, Japan). In each biological replicate, five randomly chosen, non-overlapping fields were evaluated per treatment group. Each field contained approximately 100–150 DAPI-positive nuclei, yielding an estimated 500–750 cells per condition per replicate. Images showing edge artifacts, nonuniform staining, or substantial cell aggregation were omitted. Signal intensity was analyzed with ImageJ software (version 1.53; NIH, Bethesda, MD, USA). For each field, the background-corrected integrated fluorescence was divided by the corresponding number of DAPI-positive nuclei to derive a per-cell fluorescence value. The five field-level measurements were averaged to generate one value for each biological replicate, and five independent replicates were included per condition. Acquisition parameters and threshold settings were kept constant among groups within the same experiment. Data were reported as fold change relative to the vehicle-treated control.

### 2.9. Western Blot

Total protein was isolated from treated cells using radioimmunoprecipitation assay lysis buffer (Cat# ab288006; Abcam, Cambridge, UK), and protein concentrations were measured with a BCA protein assay kit (Cat# ab102536; Abcam, Cambridge, UK). Equal amounts of protein lysates (30 μg) were separated on 10% acrylamide gels by sodium dodecyl sulfate-polyacrylamide gel electrophoresis and subsequently transferred onto polyvinylidene fluoride membranes (Cat# 03010040001; Roche, Basel, Switzerland). After blocking with 5% non-fat milk in Tris-buffered saline containing 0.1% Tween-20 (TBST) for 1 h at room temperature, the membranes were incubated overnight at 4 °C with primary antibodies against SIRT1 (Cat# ab189494), p53 (Cat# ab90363), p21 (Cat# ab109199), p16 (Cat# ab51243), or β-actin (Cat# ab8227), all purchased from Abcam (Cambridge, UK). Following TBST washes, the membranes were incubated with horseradish peroxidase-conjugated secondary antibodies for 1 h at room temperature. Protein bands were detected using enhanced chemiluminescence reagent (Cat# GERPN2209; Sigma-Aldrich, St. Louis, MO, USA) and imaged with a ChemiDoc XRS+ system (Bio-Rad, Hercules, CA, USA). Band intensities were quantified using ImageJ software (version 1.53; NIH, Bethesda, MD, USA), normalized to β-actin, and expressed as fold change relative to the vehicle-treated control group.

### 2.10. Quantitative Real-Time PCR Analysis

Total RNA was extracted from SH-SY5Y cells using RNAiso Plus (Cat# 9108; Takara Bio Inc., Shiga, Japan) following the manufacturer’s protocol. RNA quantity and purity were evaluated with a NanoDrop™ 2000 spectrophotometer (Thermo Fisher Scientific, Waltham, MA, USA), and only samples with an A260/A280 ratio of 1.8–2.0 were used. RNA integrity was further checked by agarose gel electrophoresis. For cDNA synthesis, 1 μg of total RNA was reverse-transcribed in a 20 μL reaction using the PrimeScript^®^ RT reagent kit (Cat# RR037A; Takara Bio Inc., Shiga, Japan) with oligo (dT) and random hexamer primers. The resulting cDNA was diluted tenfold with nuclease-free water and stored at −20 °C until use.

Quantitative real-time PCR was performed using SYBR^®^ Premix Ex Taq™ II (Cat# RR820A; Takara Bio Inc., Shiga, Japan) on a CFX96 Real-Time PCR Detection System (Bio-Rad Laboratories, Hercules, CA, USA). Each 20 μL reaction contained 10 μL of 2× SYBR Green master mix, 0.4 μmol/L each of forward and reverse primers, 2 μL of diluted cDNA, and nuclease-free water. The amplification conditions were as follows: initial denaturation at 95 °C for 30 s, followed by 40 cycles of denaturation at 95 °C for 5 s and annealing/extension at 60 °C for 30 s. Melting-curve analysis from 65 to 95 °C was performed to verify single-product amplification. To monitor the gene expression, the following primers were used in this study: IL-1β, forward: 5′-CTCAACTGTGAAATGCCACC-3′; reverse: 5′- GAGTGATACTGCCTGCCTGA-3′; IL-6, forward: 5′-TGGCTAAGGACCAAGACCATCCAA-3′; reverse: 5′- AACGCAC-TAGGTTTGCCGAGTAGA-3′; IL-8, forward: 5′-AGAACATCCAGAGTTTGAAGGTGAT-3′; reverse: 5′-GTGGCTATGACTTCGGTTTGG -3′. H2AX, forward: 5′-ACTCAACTCGGCAATCCAAG-3ʹ; reverse: 5′-GGGTTAGCTGCAGAATTCCA-3′. Ki67, forward: 5′- ACGCCTGGTTACTATCAAAAGG-3′; reverse: 5′- CAGACCCATTTACTTGTGTTGGA-3ʹ. SIRT1, forward: 5′-TAGACACGCTGGAACAGGTTGC-3′; reverse: 5′-CTCCTCGTACAGCTTCACAGTC-3′. p53, forward: 5′-CCTCAGCATCTTATCCGAGTGG-3′, reverse: 5′-TGGATGGTGGTACAGTCAGAGC-3′; p21, forward: 5′-AGGTGGACCTGGAGACTCTCAG-3′, reverse: 5′-TCCTCTTGGAGAAGATCAGCCG-3′; p16, forward: 5′-GGGTTTCGCCCAACGCCCCGA-3′, reverse: 5′-TGCAGCACCACCAGCGTGTCC-3′. β-actin, forward: 5′-TGTCCACCTTCCAGCAGATGT-3′, reverse: 5′-AGCTCAGTAACAGTCCGCCTAGA-3′. All reactions were performed in technical triplicate, and no-template controls were included to monitor potential contamination. Ct values were obtained using Bio-Rad CFX Manager 3.0 software. Relative gene expression was normalized to β-actin and calculated using the 2^−ΔΔCt^ method [29]. Results were expressed as fold change relative to the vehicle-treated control group.

### 2.11. SIRT1 Activity Assay

SIRT1 activity was measured using a fluorometric SIRT1 activity assay kit (Cat# ab156065; Abcam, Cambridge, UK) with minor modifications. After treatment, approximately 2 × 10^6^ cells were collected, washed with ice-cold PBS, and lysed in non-denaturing buffer to preserve enzymatic activity. Lysates were sonicated on ice and centrifuged at 12,000× *g* for 10 min at 4 °C. Equal amounts of protein were then immunoprecipitated with an anti-SIRT1 antibody (Cat# ab12193; Abcam, Cambridge, UK) and Protein A agarose beads. The SIRT1 immunoprecipitates were incubated with assay buffer, fluorogenic substrate, NAD^+^, and developer in black 96-well plates. Fluorescence was monitored at 350/460 nm every 1–2 min for 30–60 min using a SpectraMax M5 microplate reader (Molecular Devices, Sunnyvale, CA, USA). Enzymatic activity was calculated from the linear portion of the fluorescence curve after subtraction of the no-enzyme background. Values were normalized to the vehicle-treated control group and expressed as a percentage of the control.

### 2.12. Statistical Analysis

All data are presented as the mean ± standard deviation (SD). Each experiment was performed using five independent biological replicates (*n* = 5), with technical triplicates obtained for each treatment condition. Technical triplicates were averaged before statistical analysis, and biological replicate values were used for group comparisons. Statistical analyses were conducted using SigmaPlot (version 14; Systat Software, Inc., San Jose, CA, USA). For the Aβ_1–42_ oligomer concentration–time viability assay, comparisons among the vehicle, 5, 10, and 20 μmol/L Aβ_1–42_ oligomer groups were performed separately at each fixed incubation time using one-way analysis of variance (ANOVA), followed by Tukey’s multiple comparisons test. Other comparisons involving more than two groups were analyzed using one-way ANOVA followed by Tukey’s multiple comparisons test. Comparisons between two groups were performed using a two-tailed Student’s t-test. A *p*-value < 0.05 was considered statistically significant.

## 3. Results

### 3.1. PCB Attenuated Aβ_1–42_ Oligomer-Induced Cytotoxicity in SH-SY5Y Cells

**Figure 1:**
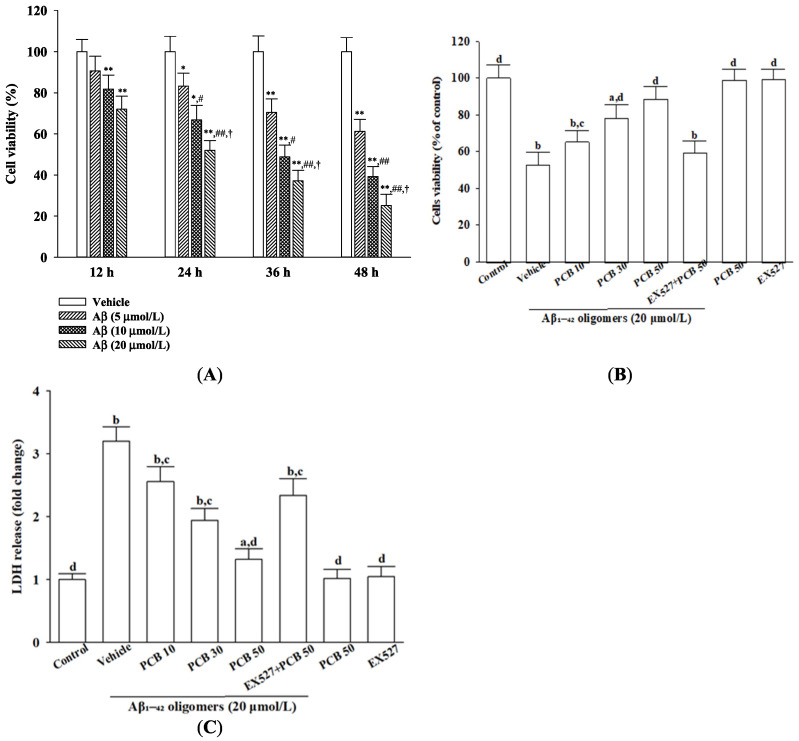
Protective effects of phycocyanobilin (PCB) against amyloid-β (Aβ)_1–42_ oligomer-induced cytotoxicity in SH-SY5Y cells. (**A**) Effects of Aβ_1–42_ oligomers on cell viability. SH-SY5Y cells were treated with Aβ_1–42_ oligomers at 5, 10, or 20 μmol/L for 12, 24, 36, or 48 h, and cell viability was determined using the CCK-8 assay. (**B**) Effects of PCB on Aβ_1–42_ oligomer-induced cytotoxicity. Cells were pretreated with PCB at 10, 30, or 50 μmol/L for 24 h, followed by exposure to Aβ_1–42_ oligomers at 20 μmol/L for an additional 24 h. Where indicated, cells were co-pretreated with PCB and EX527, a selective SIRT1 inhibitor used at 10 μmol/L, before exposure to Aβ_1–42_ oligomers. Cell viability was then assessed using the CCK-8 assay. (**C**) Effects of PCB on Aβ_1–42_ oligomer-induced lactate dehydrogenase (LDH) release. Cells were treated as described in (**B**), and LDH release was measured as an indicator of membrane damage and cytotoxicity. Data are presented as the mean ± SD from five independent biological replicates (*n* = 5). Technical triplicates were averaged to obtain one value for each biological replicate before statistical analysis. Statistical comparisons among the vehicle, 5, 10, and 20 μmol/L Aβ_1–42_ oligomer groups were performed separately at each fixed incubation time using one-way ANOVA followed by Tukey’s multiple comparisons test. * *p* < 0.05 and ** *p* < 0.01 vs. the vehicle group at the corresponding incubation time; ^#^ *p* < 0.05 and ^##^ *p* < 0.01 vs. the 5 μmol/L Aβ_1–42_ oligomer group at the corresponding incubation time; ^†^ *p* < 0.05 vs. the 10 μmol/L Aβ_1–42_ oligomer group at the corresponding incubation time. Data in Figure 1B,C were analyzed by one-way ANOVA followed by Tukey’s post hoc test. For Figure 1B,C: ^a^ *p* < 0.05, ^b^ *p* < 0.01 vs. vehicle-treated control cells; ^c^ *p* < 0.05, ^d^ *p* < 0.01 vs. vehicle-treated cells exposed to Aβ_1–42_ oligomers alone.

As shown in Figure 1A, exposure to Aβ_1–42_ oligomers reduced SH-SY5Y cell viability in a concentration- and time-dependent manner. Vehicle-treated cells maintained viability close to 100% throughout the experimental period. The strongest cytotoxic effect was observed at 20 μmol/L Aβ_1–42_ oligomers, which reduced cell viability to approximately 50–55% after 24 h and further decreased viability to approximately 25–30% after 48 h. Therefore, 20 μmol/L Aβ_1–42_ oligomers for 24 h was selected as the standard injury condition for subsequent experiments because this condition produced moderate, reproducible cytotoxicity while retaining sufficient viable cells for evaluating PCB-mediated protection.

As shown in Figure 1B, PCB pretreatment restored the reduction in cell viability induced by 20 μmol/L Aβ_1–42_ oligomers in a concentration-dependent manner. PCB at 10, 30, and 50 μmol/L increased cell viability to approximately 65%, 78%, and nearly 90% of the control level, respectively. Because 50 μmol/L PCB produced the strongest cytoprotective effect without causing cytotoxicity when administered alone, this concentration was defined as the effective working concentration and used for the subsequent mechanistic and senescence-related experiments. Co-treatment with the SIRT1 inhibitor EX527 partially reduced the protective effect of PCB, lowering cell viability to approximately 60%. In contrast, treatment with PCB alone at 50 μmol/L or EX527 alone at 10 μmol/L did not significantly affect cell viability compared with the untreated control group.

Consistent with the cell viability results, LDH release analysis showed that exposure to 20 μmol/L Aβ_1–42_ oligomers markedly increased cytotoxicity-associated membrane damage (Figure 1C). Aβ_1–42_ oligomer treatment increased LDH release to approximately 3.2-fold relative to the control group. PCB pretreatment reduced Aβ_1–42_ oligomer-induced LDH release in a concentration-dependent manner, with LDH release decreasing to approximately 2.5-, 1.9-, and 1.3-fold of the control level following treatment with 10, 30, and 50 μmol/L PCB, respectively. However, EX527 co-treatment partially reversed the inhibitory effect of PCB on LDH release, increasing LDH release to approximately 2.3-fold of the control level. PCB alone and EX527 alone did not noticeably alter LDH release, with values remaining close to those of the control group.

### 3.2. PCB Attenuated Aβ_1–42_ Oligomer-Induced Cellular Senescence and Associated Inflammatory Responses

**Figure 2:**
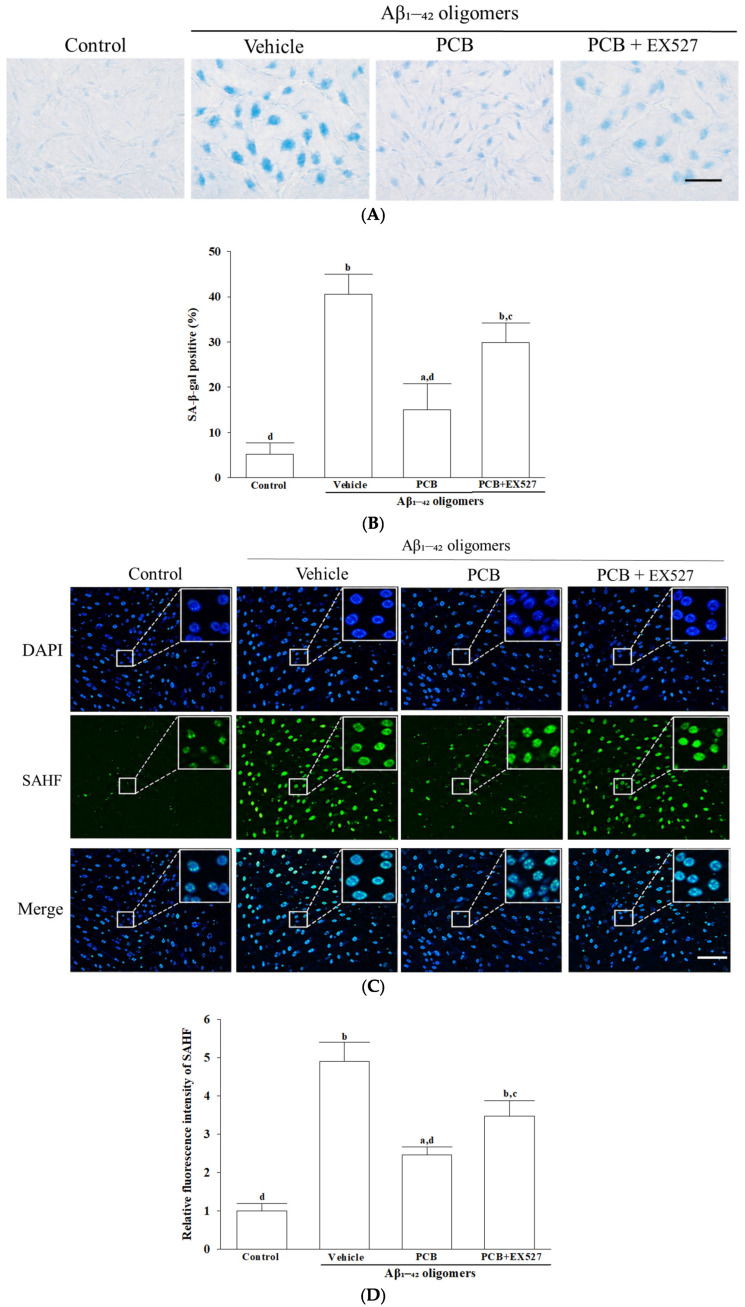

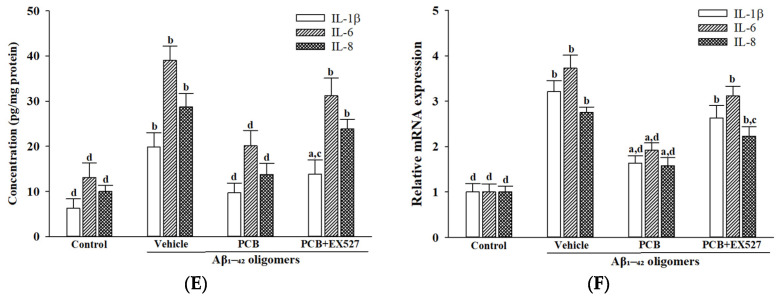
Phycocyanobilin (PCB) attenuates amyloid-β (Aβ)_1–42_ oligomer-induced cellular senescence and senescence-associated secretory phenotype (SASP)-associated inflammatory responses in SH-SY5Y cells. Cells were pretreated with PCB at 50 μmol/L in the presence or absence of the SIRT1 inhibitor EX527 at 10 μmol/L for 24 h, followed by exposure to Aβ_1–42_ oligomers at 20 μmol/L for an additional 24 h. (**A**) Representative images of senescence-associated β-galactosidase (SA-β-gal) staining under different treatment conditions. Blue staining indicates SA-β-gal-positive senescent cells. Original magnification: 100×; scale bar: 100 μm. (**B**) Quantitative analysis of the percentage of SA-β-gal-positive cells. (**C**) Representative immunofluorescence images of senescence-associated heterochromatin foci (SAHF) formation. Nuclei were counterstained with DAPI (blue), and SAHF-associated di-/tri-methylated histone H3 lysine 9 (H3K9me2/3) signals are shown in green. Original magnification: 100×; scale bar: 100 μm. The boxed areas in the full-field images are shown as enlarged insets to facilitate visualization of the nuclear staining patterns. Insets were generated from the corresponding original images without differential adjustment of fluorescence intensity between treatment groups. (**D**) Quantitative analysis of the relative fluorescence intensity of SAHF-associated H3K9me2/3. (**E**) Concentrations of the SASP-associated inflammatory cytokines IL-1β, IL-6, and IL-8 in culture supernatants. (**F**) Relative mRNA expression levels of interleukin (IL)-1β, IL-6, and IL-8. Data are presented as the mean ± SD from five independent biological replicates (*n* = 5). Technical triplicates were averaged before statistical analysis. Statistical significance was determined by one-way ANOVA followed by Tukey’s post hoc test. ^a^ *p* < 0.05, ^b^ *p* < 0.01 vs. vehicle-treated control cells (control); ^c^ *p* < 0.05, ^d^ *p* < 0.01 vs. vehicle-treated cells exposed to Aβ_1–42_ oligomers (20 μmol/L) alone.

As shown in Figure 2A, exposure to 20 μmol/L Aβ_1–42_ oligomers markedly increased SA-β-gal staining in SH-SY5Y cells, indicating the induction of a senescence-like phenotype. Quantitative analysis showed that the percentage of SA-β-gal-positive cells increased from approximately 5% in the control group to approximately 40% in the Aβ_1–42_ oligomer-treated vehicle group (Figure 2B). PCB pretreatment at 50 μmol/L significantly reduced the proportion of SA-β-gal-positive cells induced by Aβ_1–42_ oligomers to approximately 15%. However, co-treatment with EX527 at 10 μmol/L partially abolished the anti-senescent effect of PCB, increasing the percentage of SA-β-gal-positive cells to approximately 30% (Figure 2B).

Consistent with the SA-β-gal results, exposure to 20 μmol/L Aβ_1–42_ oligomers markedly increased SAHF-associated heterochromatin remodeling, as reflected by enhanced H3K9me2/3-associated fluorescence staining (Figure 2C). Quantitative analysis showed that exposure to Aβ_1–42_ oligomers increased the relative fluorescence intensity of SAHF-associated signals to approximately 4.8-fold of the control level, whereas pretreatment with 50 μmol/L PCB significantly attenuated this increase, reducing the fluorescence intensity to approximately 2.5-fold of the control level (Figure 2D). In contrast, co-treatment with EX527 (10 μmol/L) partially reversed the inhibitory effect of PCB (50 μmol/L), increasing the fluorescence intensity to approximately 3.5-fold of the control level (Figure 2D).

Moreover, Aβ_1–42_ oligomers markedly induced inflammatory responses, as evidenced by significant increases in both the protein concentrations (Figure 2E) and mRNA expression levels (Figure 2F) of IL-1β, IL-6, and IL-8. At the protein level, exposure to 20 μmol/L Aβ_1–42_ oligomers increased IL-1β, IL-6, and IL-8 levels to approximately 3.3-, 3.3-, and 2.9-fold of the control level, respectively (Figure 2E). Pretreatment with 50 μmol/L PCB attenuated these increases, reducing IL-1β, IL-6, and IL-8 levels to approximately 1.7-, 1.7-, and 1.4-fold of the control level, respectively. How-ever, this inhibitory effect was partially reversed by co-treatment with 10 μmol/L EX527, with IL-1β, IL-6, and IL-8 levels increasing to approximately 2.3-, 2.6-, and 2.4-fold of the control level, respectively (Figure 2E).

A similar trend was observed at the transcriptional level. Aβ_1–42_ oligomers (20 μmol/L) exposure increased IL-1β, IL-6, and IL-8 mRNA expression to approximately 3.2-, 3.7-, and 2.8-fold of the control level, respectively (Figure 2F). PCB (50 μmol/L) pretreatment markedly suppressed these elevations, reducing IL-1β, IL-6, and IL-8 mRNA expression to approximately 1.6-, 1.9-, and 1.5-fold of the control level, respectively. In contrast, EX527 (10 μmol/L) co-treatment partially reversed the suppressive effect of PCB (50 μmol/L), increasing IL-1β, IL-6, and IL-8 mRNA expression to approximately 2.6-, 3.1-, and 2.2-fold of the control level, respectively (Figure 2F).

### 3.3. PCB Attenuated Aβ_1–42_-Induced DNA Damage in Neuronal Cells

**Figure 3:**
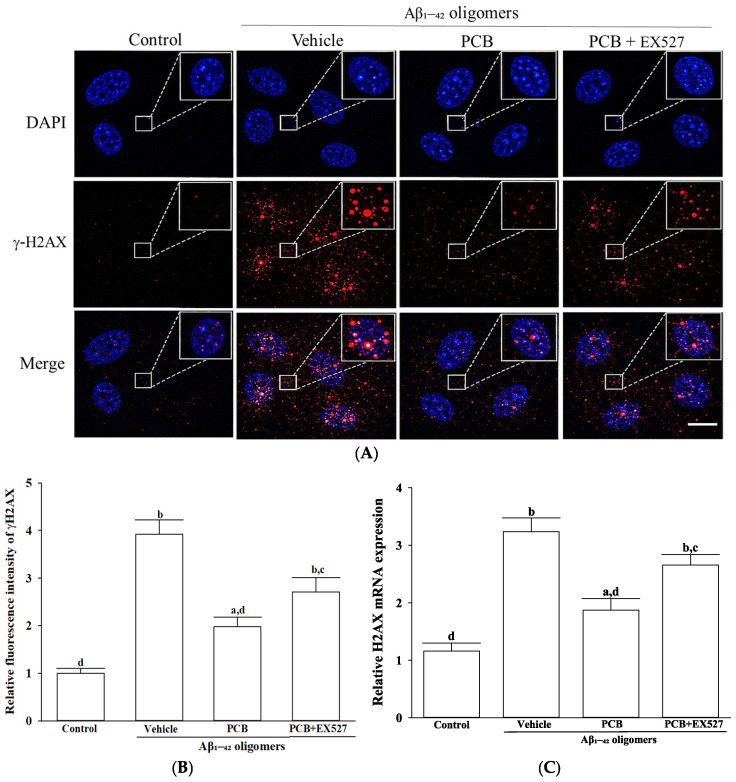
Phycocyanobilin (PCB) attenuates amyloid-β (Aβ)_1–42_ oligomer-induced DNA damage in SH-SY5Y cells. Cells were pretreated with PCB at 50 μmol/L in the presence or absence of the SIRT1 inhibitor EX527 at 10 μmol/L for 24 h, followed by exposure to Aβ_1–42_ oligomers at 20 μmol/L for an additional 24 h. (**A**) Immunofluorescence staining of phosphorylated histone H2AX (γ-H2AX) was performed to evaluate DNA damage. Representative images of DAPI-stained nuclei (blue), γ-H2AX signals (red), and merged images are shown. Original magnification: 100×; scale bar: 100 μm. The boxed areas in the full-field images are shown as enlarged insets to facilitate visualization of the nuclear staining patterns. Insets were generated from the corresponding original images without differential adjustment of fluorescence intensity between treatment groups. (**B**) Quantitative analysis of γ-H2AX fluorescence intensity is expressed as relative fluorescence intensity normalized to the control group. (**C**) Relative H2AX mRNA expression levels determined by quantitative real-time polymerase chain reaction and normalized to the control group. Data are presented as the mean ± SD from five independent biological replicates (*n* = 5). Technical triplicates were averaged before statistical analysis. Statistical significance was determined by one-way ANOVA followed by Tukey’s post hoc test. ^a^ *p* < 0.05, ^b^ *p* < 0.01 vs. vehicle-treated control cells (control); ^c^ *p* < 0.05, ^d^ *p* < 0.01 vs. vehicle-treated cells exposed to Aβ_1–42_ oligomers (20 μmol/L) alone.

As shown in the immunofluorescence images (Figure 3A), the vehicle-treated control group displayed only faint, negligible γ-H2AX signals. In contrast, exposure to Aβ_1–42_ oligomers (20 μmol/L) triggered a robust accumulation of γ-H2AX foci within the nuclei. Pretreatment with PCB (50 μmol/L) substantially reduced Aβ_1–42_ oligomer-induced γ-H2AX accumulation, whereas co-treatment with EX527 (10 μmol/L) partially reversed this protective effect.

Quantitative analysis (Figure 3B) revealed that the relative fluorescence intensity surged to approximately 3.9-fold of the control level (*p* < 0.05). Pretreatment with PCB (50 μmol/L) reduced γ-H2AX accumulation induced by Aβ_1–42_ oligomers (20 μmol/L), with fluorescence intensity decreasing to approximately 2.0-fold, representing a reduction of nearly 50% compared with the Aβ_1–42_ oligomer (20 μmol/L)-treated group. However, co-treatment with EX527 (10 μmol/L) partially abolished the protective effect of PCB (50 μmol/L), as γ-H2AX fluorescence intensity increased to approximately 2.7-fold of the control level, which was higher than that observed with PCB pretreatment alone but still significantly lower than that in cells treated with Aβ_1–42_ oligomers (20 μmol/L) alone (*p* < 0.05).

Consistently, exposure to Aβ_1–42_ oligomers (20 μmol/L) also increased H2AX mRNA expression to approximately 3.2-fold of the control level (Figure 3C). PCB pretreatment (50 μmol/L) reduced H2AX mRNA expression to approximately 1.9-fold of the control level, whereas co-treatment with EX527 (10 μmol/L) partially reversed this effect, increasing H2AX mRNA expression to approximately 2.6-fold of the control level.

### 3.4. PCB Attenuated Aβ_1–42_ Oligomer-Induced Suppression of Ki67 Expression in SH-SY5Y Cells

**Figure 4:**
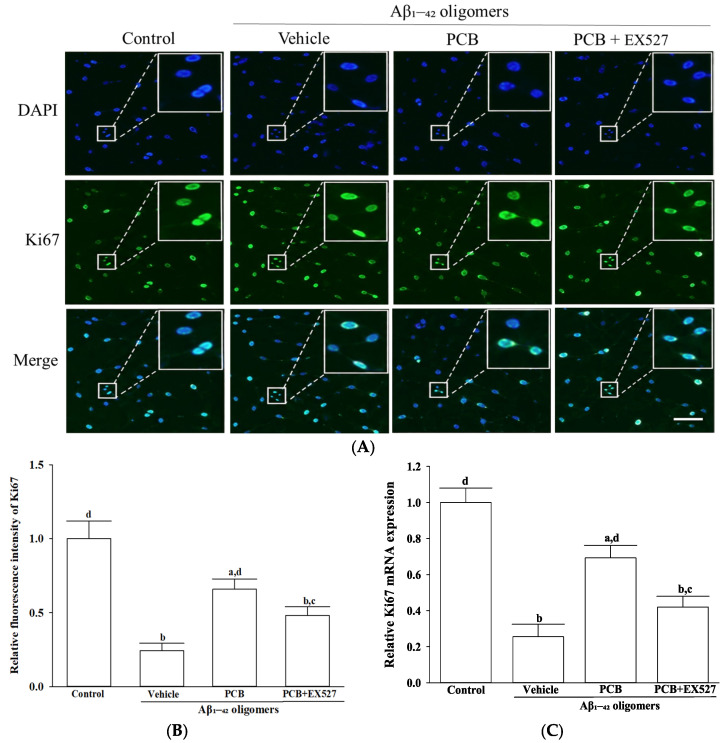
Phycocyanobilin (PCB) attenuates amyloid-β (Aβ)_1–42_ oligomer-induced suppression of Ki67 expression in SH-SY5Y cells. Cells were pretreated with PCB at 50 μmol/L in the presence or absence of the SIRT1 inhibitor EX527 at 10 μmol/L for 24 h, followed by exposure to Aβ_1–42_ oligomers at 20 μmol/L for an additional 24 h. (**A**) Representative immunofluorescence images of Ki67 staining. Nuclei were stained with DAPI (blue), and Ki67-positive signals are shown in green. Merged images are shown. Images were captured using a fluorescence microscope (original magnification: 100×; scale bar = 100 μm). The boxed areas in the full-field images are shown as enlarged insets to facilitate visualization of the nuclear staining patterns. Insets were generated from the corresponding original images without differential adjustment of fluorescence intensity between treatment groups. (**B**) Quantitative analysis of Ki67 fluorescence intensity is expressed as relative fluorescence intensity normalized to the control group. (**C**) Relative Ki67 mRNA expression levels determined by quantitative real-time polymerase chain reaction and normalized to the control group. Data are presented as the mean ± SD from five independent biological replicates (*n* = 5). Technical triplicates were averaged before statistical analysis. Statistical significance was determined by one-way ANOVA followed by Tukey’s post hoc test. ^a^ *p* < 0.05, ^b^ *p* < 0.01 vs. vehicle-treated control cells (control); ^c^ *p* < 0.05, ^d^ *p* < 0.01 vs. vehicle-treated cells exposed to Aβ_1–42_ oligomers (20 μmol/L) alone.

As shown in Figure 4A, immunofluorescence staining demonstrated that exposure to Aβ_1–42_ oligomers (20 μmol/L) markedly reduced Ki67-positive fluorescence signals compared with the untreated control group, suggesting suppression of a cell-cycle-associated marker in differentiated neuron-like SH-SY5Y cells. Pretreatment with PCB at 50 μmol/L partially restored Ki67 fluorescence in Aβ_1–42_ oligomer-exposed cells, whereas co-treatment with EX527 at 10 μmol/L attenuated this restorative effect.

Analysis of Ki67 mRNA expression (Figure 4C) revealed a downregulation following Aβ_1–42_ oligomer (20 μmol/L) exposure, to approximately 25% of the control group (*p* < 0.05). PCB (50 μmol/L) pretreatment increased Ki67 mRNA expression to approximately 70% of the control level, representing an approximately 2.6-fold increase compared with the Aβ_1–42_ oligomer-treated group (*p* < 0.05). Co-treatment with EX527 (10 μmol/L) attenuated the PCB (50 μmol/L)-mediated restoration of Ki67 mRNA expression, reducing it to approximately 60% of that in the PCB (50 μmol/L)-pretreated group (*p* < 0.05).

### 3.5. PCB Attenuated Aβ_1–42_ Oligomer-Induced Upregulation of Senescence-Related Markers

**Figure 5:**
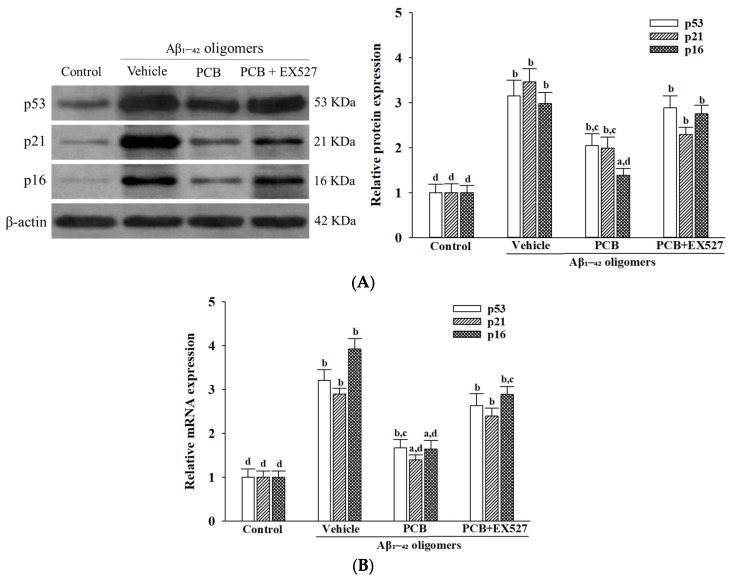
Phycocyanobilin (PCB) attenuates amyloid-β (Aβ)_1–42_ oligomer-induced upregulation of senescence-related markers in SH-SY5Y cells. Cells were pretreated with PCB at 50 μmol/L in the presence or absence of the SIRT1 inhibitor EX527 at 10 μmol/L for 24 h, followed by exposure to Aβ_1–42_ oligomers at 20 μmol/L for an additional 24 h. (**A**) Representative Western blot images showing the expression levels of p53, p21, and p16, with β-actin used as the internal loading control. (**B**) Relative mRNA expression levels of p53, p21, and p16 determined by quantitative real-time polymerase chain reaction and normalized to β-actin. Data are presented as the mean ± SD from five independent biological replicates (*n* = 5). Technical triplicates were averaged before statistical analysis. Statistical significance was determined by one-way ANOVA followed by Tukey’s post hoc test. ^a^ *p* < 0.05, ^b^ *p* < 0.01 vs. vehicle-treated control cells (control); ^c^ *p* < 0.05, ^d^ *p* < 0.01 vs. vehicle-treated cells exposed to Aβ_1–42_ oligomers (20 μmol/L) alone.

As shown in Figure 5A, exposure to Aβ_1–42_ oligomers (20 μmol/L) markedly upregulated the protein expression of the senescence-related markers p53, p21, and p16 in SH-SY5Y cells. Densitometric analysis revealed that Aβ_1–42_ oligomers (20 μmol/L) exposure increased p53, p21, and p16 protein levels to approximately 3.1-, 3.4-, and 3.0-fold of the control level, respectively. Pretreatment with PCB at 50 μmol/L substantially attenuated these increases, reducing p53, p21, and p16 protein expression to approximately 2.0-, 2.0-, and 1.4-fold of the control level, respectively. However, co-treatment with EX527 at 10 μmol/L partially reversed the inhibitory effect of PCB (50 μmol/L), resulting in re-elevation of p53, p21, and p16 protein levels to approximately 2.9-, 2.3-, and 2.7-fold of the control level, respectively.

Consistent with the protein expression results, qRT-PCR analysis showed that Aβ_1–42_ oligomer exposure (20 μmol/L) increased p53, p21, and p16 mRNA expression to approximately 3.2-, 2.9-, and 3.9-fold of the control level, respectively (Figure 5B). PCB (50 μmol/L) pretreatment markedly reduced these elevations, lowering p53, p21, and p16 mRNA expression to approximately 1.6-, 1.4-, and 1.6-fold of the control level, respectively. In contrast, EX527 (10 μmol/L) co-treatment partially abolished the suppressive effect of PCB, increasing p53, p21, and p16 mRNA expression to approximately 2.6-, 2.4-, and 2.9-fold of the control level, respectively.

### 3.6. PCB Restored SIRT1 Expression and Enzymatic Activity in Aβ_1–42_ Oligomer-Exposed SH-SY5Y Cells

**Figure 6:**
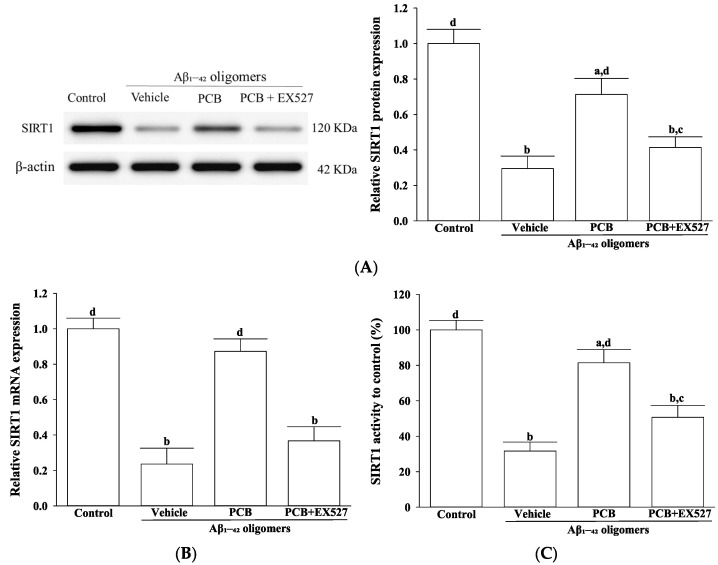
Phycocyanobilin (PCB) restored silent information regulator 1 (SIRT1) expression and enzymatic activity in amyloid-β (Aβ)_1–42_ oligomer-exposed SH-SY5Y cells. Cells were pretreated with PCB at 50 μmol/L in the presence or absence of the SIRT1 inhibitor EX527 at 10 μmol/L for 24 h, followed by exposure to Aβ_1–42_ oligomers at 20 μmol/L for an additional 24 h. (**A**) Representative Western blot images of SIRT1 protein expression and densitometric quantification normalized to β-actin. (**B**) Relative SIRT1 mRNA expression levels determined by quantitative real-time polymerase chain reaction and normalized to β-actin. (**C**) SIRT1 enzymatic activity assessed using a fluorometric activity assay and expressed as a percentage relative to the control group. Data are presented as the mean ± SD from five independent biological replicates (*n* = 5). Technical triplicates were averaged before statistical analysis. Statistical significance was determined by one-way ANOVA followed by Tukey’s post hoc test. ^a^ *p* < 0.05, ^b^ *p* < 0.01 vs. vehicle-treated control cells (control); ^c^ *p* < 0.05, ^d^ *p* < 0.01 vs. vehicle-treated cells exposed to Aβ_1–42_ oligomers (20 μmol/L) alone.

As shown in Figure 6A, exposure to Aβ_1–42_ oligomers (20 μmol/L) suppressed SIRT1 protein expression, reducing it to approximately 30% of the control level. Pretreatment with PCB at 50 μmol/L partially restored SIRT1 protein expression under Aβ_1–42_ oligomer (20 μmol/L) exposure, increasing it to approximately 70% of the control level. However, co-treatment with EX527 at 10 μmol/L attenuated this PCB-mediated restoration, reducing SIRT1 protein expression to approximately 40% of the control level.

A similar trend was observed for SIRT1 mRNA expression (Figure 6B). Aβ_1–42_ oligomers (20 μmol/L) treatment decreased SIRT1 transcript levels to approximately 25% of the control level, whereas PCB (50 μmol/L) pretreatment increased SIRT1 mRNA expression to approximately 85% of the control level under Aβ_1–42_ oligomers (20 μmol/L) exposure. However, EX527 (10 μmol/L) co-treatment attenuated this PCB (50 μmol/L)-mediated increase, lowering SIRT1 mRNA expression to approximately 35% of the control level.

Consistent with these changes in SIRT1 expression, SIRT1 enzymatic activity was also reduced following exposure to Aβ_1–42_ oligomers (20 μmol/L), declining to approximately 30% of the control level (Figure 6C). PCB (50 μmol/L) pretreatment restored SIRT1 activity to approximately 80% of the control level, whereas EX527 (10 μmol/L) co-treatment significantly diminished this effect, reducing SIRT1 activity to approximately 50% of the control level.

## 4. Discussion

The present study extends the neuroprotective profile of PCB by demonstrating its ability to attenuate Aβ_1–42_ oligomer-induced neuronal senescence, a pathological process distinct from the glycation-associated neuronal injury model previously investigated [17]. The use of Aβ_1–42_ oligomers is particularly relevant because soluble Aβ oligomeric species are among the most neurotoxic forms of Aβ, closely linked to synaptic dysfunction, oxidative stress, inflammatory activation, and neuronal vulnerability in AD [18]. Compared with insoluble fibrillar deposits, Aβ oligomers better reflect the early and dynamic proteotoxic stress that may initiate neuronal dysfunction before extensive plaque formation [18]. Therefore, an Aβ_1–42_ oligomer-induced injury model provides a biologically meaningful platform for examining whether PCB can counteract AD-related neuronal stress beyond glycation-associated toxicity. This distinction is important because Aβ oligomers represent a core AD-related proteotoxic trigger, whereas cellular senescence provides a mechanistic framework through which transient Aβ-induced injury may evolve into persistent neuroinflammatory and degenerative signaling [30]. In this context, our findings identify Aβ-induced neuronal senescence as a new mechanistic setting for PCB action and broaden the biological relevance of PCB beyond its previously recognized antioxidative and cytoprotective properties [16,17]. Given that cellular senescence has increasingly been implicated as a pathological amplifier in AD, targeting the Aβ oligomer–senescence axis may offer a complementary strategy beyond approaches focused solely on amyloid burden reduction.

A major strength of this study is the use of multiple complementary senescence-associated endpoints to assess the effects of PCB. Cellular senescence is a multifaceted biological state that cannot be defined by a single marker, but instead reflects the integration of lysosomal alterations, chromatin remodeling, DNA damage responses, inflammatory secretion, and cell-cycle regulatory changes [30]. On this basis, the present study examined a broad panel of senescence-related markers, including SA-β-gal activity, SAHF-associated H3K9me2/3 signals, SASP-related cytokine production, γ-H2AX-associated DNA damage, Ki67 expression, and the canonical p53/p21 and p16 signaling pathways. This approach allowed us to evaluate phenotypic, inflammatory, DNA damage-related, and cell-cycle regulatory aspects of Aβ-induced senescence-like alterations in a more comprehensive manner [31]. It is important, however, to interpret Ki67 expression cautiously in this experimental context. Differentiated SH-SY5Y cells acquire neuron-like characteristics and are not actively proliferating in the same manner as dividing cell populations. Therefore, changes in Ki67 should not be regarded as direct evidence of altered proliferative capacity. Instead, Ki67 was included as a supportive cell-cycle-associated marker within a broader senescence-related marker panel. The coordinated attenuation of these endpoints by PCB suggests that PCB does not merely affect a single downstream marker, but may interfere with a broader Aβ-induced senescence-like network. In particular, the reduction in SASP-associated cytokines, including IL-1β, IL-6, and IL-8, is mechanistically relevant because these inflammatory mediators may reinforce neuroinflammation, promote secondary senescence, and sustain neuronal vulnerability within AD-related microenvironments [9]. Taken together, these findings support the interpretation that PCB may act as an anti-senescent modulator targeting the interconnected damage–inflammation–cell-cycle dysregulation axis induced by Aβ oligomers, thereby providing a stronger mechanistic rationale for its potential relevance to AD-related neuronal aging.

SIRT1 is a stress-responsive NAD^+^-dependent deacetylase that regulates genomic stability, DNA repair, mitochondrial homeostasis, inflammatory responses, metabolic adaptation, and senescence-associated signaling [12,13]. In AD-related neuronal aging, impaired SIRT1 activity may increase cellular vulnerability to Aβ-induced oxidative stress, DNA damage, inflammatory activation, and senescence-like growth arrest [32]. Therefore, restoration of SIRT1 signaling by natural products may represent a potential strategy for limiting the progression from acute Aβ-associated injury to sustained neuronal dysfunction [33,34]. In the present study, PCB restored SIRT1 expression and activity, which may help explain the coordinated attenuation of γ-H2AX accumulation, p53/p21/p16 signaling, SASP-related cytokine production, SA-β-gal positivity, SAHF-associated H3K9me2/3 signals, and suppression of Ki67 expression. The partial reversal of these protective effects by EX527, a selective SIRT1 inhibitor, further supports the functional involvement of SIRT1-associated mechanisms in PCB-mediated protection [18]. However, EX527 did not completely abolish the effects of PCB, indicating that PCB should not be interpreted as a selective SIRT1 activator or as acting exclusively through SIRT1. Rather, PCB likely functions as a pleiotropic stress-modulating compound whose actions are partly mediated by SIRT1-associated regulation and partly related to SIRT1-independent mechanisms, including intrinsic antioxidant activity, suppression of ROS-generating pathways, attenuation of inflammatory signaling, preservation of mitochondrial function, regulation of redox-sensitive pathways, and reduction in DNA damage responses. This interpretation is consistent with the multifactorial nature of Aβ-induced neuronal injury, which involves interconnected cytotoxic, oxidative, inflammatory, DNA damage-related, and senescence-associated processes [7]. Accordingly, the present findings support an anti-senescence-associated or senescence-modulating effect of PCB under Aβ_1–42_-induced neuronal stress, while also indicating that these effects may partly result from broader cytoprotective and stress-reducing actions rather than a direct anti-senescent mechanism independent of upstream injury attenuation.

The present study extends previous PCB-related research, which primarily described PCB as a functional food-derived compound capable of mitigating glycation-associated neuronal injury [17]. By moving the pathological focus from metabolic/glycation stress to Aβ_1–42_ oligomer-evoked neuronal senescence, our findings broaden the mechanistic context in which this algae-derived compound may exert neuroprotective actions. Although these two stress paradigms are distinct, both are relevant to neurodegeneration and may converge on common downstream events, including oxidative injury, disrupted cellular homeostasis, inflammatory activation, mitochondrial dysfunction, and reduced neuronal resilience [35]. In particular, Aβ_1–42_ oligomers constitute a central AD-related proteotoxic stimulus, whereas cellular senescence may act as a persistent stress-amplifying state that maintains DNA damage responses, mitochondrial impairment, inflammatory signaling, and neuronal vulnerability even after the initiating insult has occurred [8]. Thus, the protective effects observed in the present Aβ-induced senescence model suggest that PCB may have biological relevance beyond antioxidative and anti-apoptotic cytoprotection, supporting its potential role as an anti-senescence-associated modulator in AD-related neuronal aging. This view is consistent with the growing interest in algae-derived bioactive compounds as multi-mechanism neuroprotective agents [15]. Given that AD pathology involves interconnected networks of oxidative stress, inflammation, mitochondrial dysfunction, proteostasis failure, and impaired stress adaptation, compounds capable of influencing several related pathways may offer theoretical advantages over narrowly acting agents [7]. Accordingly, PCB may be better regarded as a biologically active nutritive compound that reinforces endogenous stress-adaptive mechanisms and reduces neuronal vulnerability, rather than as a single-target therapeutic molecule.

Several limitations should be acknowledged. First, although differentiated SH-SY5Y cells provide a useful neuron-like model for mechanistic investigation, they cannot fully recapitulate the biological complexity of the aging brain, including neuron–glia interactions, blood–brain barrier regulation, immune responses, vascular influences, and systemic metabolism [36]. In addition, the Aβ_1–42_ exposure paradigm used in this study represents an acute in vitro stress condition rather than a direct reproduction of physiological or pathological Aβ levels in the human brain. The concentration of 20 μmol/L was selected because it produced moderate and reproducible cytotoxicity while preserving sufficient viable cells for evaluating PCB-mediated protection. Therefore, the translational relevance of this model should be interpreted with caution, and future studies using lower and chronic Aβ exposure paradigms, three-dimensional cultures, neuron–glia co-cultures, and animal models are needed. Second, the aggregation state of the Aβ_1–42_ preparation was not directly characterized. Although Aβ_1–42_ was prepared using a previously reported oligomerization protocol and used freshly to minimize variability, biochemical or structural confirmation was not performed. Thus, the preparation should be interpreted as an oligomerization protocol-derived Aβ_1–42_ preparation rather than a biochemically verified oligomeric preparation. Finally, this study primarily evaluated PCB as a pretreatment, supporting a preventive or protective effect rather than therapeutic reversal of established pathology. Because PCB also improved cell viability and reduced LDH release, the attenuation of senescence-associated markers may partly reflect reduced upstream cytotoxic stress rather than a senescence-specific effect. Although EX527 supports the involvement of SIRT1-associated mechanisms, additional SIRT1 knockdown, overexpression, or rescue experiments are needed to strengthen causal interpretation. Moreover, the effective in vitro concentration of PCB should not be directly extrapolated to achievable brain exposure in vivo without pharmacokinetic and biodistribution studies.

## 5. Conclusions

In conclusion, this study establishes Aβ_1–42_ oligomer-induced neuronal senescence as a previously underexplored pathological context for PCB-mediated neuroprotection and highlights SIRT1-associated regulation as an important mechanistic contributor. By extending the role of PCB from general cytoprotection to the modulation of senescence-associated neuronal stress, these findings provide new mechanistic insight into PCB as a potential anti-senescent neuroprotective candidate for AD-related neuronal aging and senescence-associated neurodegeneration.

## Data Availability

The original contributions presented in this study are included in the article. Further inquiries can be directed to the corresponding authors.
